# Chagas Disease, Up Close and Personal: A Review of *The Kissing Bug* by Daisy Hernandez And a Conversation with Cardiologist Sheba Meymandi

**DOI:** 10.4269/ajtmh.22-0003

**Published:** 2022-03-14

**Authors:** Claire Panosian Dunavan

**Affiliations:** UCLA Division of Infectious Diseases CHS 52-215, 10833 Le Conte Avenue Los Angeles, California E-mail: cpanosian@mednet.ucla.edu

*Miguel never recalled when they actually began—those feeble, quivering heartbeats that snaked through his chest like a high-speed train. The first attacks were brief and he forgot them as soon as they passed. Then, over time, they came more often and lasted longer, until one day at work a friend saw the whole crazy business, the sweat on his face and the fear in his eyes. He thought Miguel was dizzy and made him lie down. Soon after, Miguel’s wife arrived and took him to the Emergency Room at L.A. County Hospital, where they did an electrocardiogram, his first ever. “You have extra heartbeats,” the nurse said, “but no sign of a heart attack.” “You’re 38 years old, your blood pressure’s great, you don’t even smoke,” the intern added. “You’ve got a lot of miles on that heart.” So Miguel decided it was nothing, and, from then on, he pretended he was fine.*

*The following winter Miguel caught a cold from his daughter. Soon his feet and ankles began to swell, and he couldn’t get air. Finally he spent an entire night bolt upright (since he could no longer catch his breath lying down), wheezing, coughing, and gurgling like a worn-out radiator. That night he also worried. He could no longer fool himself. Something was terribly wrong.*

In the mid-1990s, I met a 40-year-old man in my hospital’s Coronary Observation Unit. After two years of failing health, Miguel was awaiting a (possible) cardiac transplant. At the same time, what had caused his heart to fail in the first place was still unknown.

In a way, this wasn’t surprising. A few years earlier, cardiologists at Los Angeles’s largest public hospital hypothesized that people like Miguel could easily be misdiagnosed or lost in a sea of sufferers with more conventional ills. Writing in *The New England Journal of Medicine*, they described 25 *Trypanosoma cruzi*-infected patients whose heart disease had long been wrongly linked to atherosclerosis or labeled as a “cardiomyopathy of unknown cause.”

Like a good 30% of people carrying *T. cruzi*, Miguel’s story did not end well. He did not choose to battle his long-term protozoan foe. After we proved his diagnosis, he simply declined the transplant and went home to die. Afterward, I shared his story in *Discover* magazine.

In 2022, could another Miguel with “Chagas disease” practically blazoned on his chest still elude diagnosis until arriving on death’s door at a university hospital? Of course. Despite better serological tests and imaging as well as implanted defibrillators, left-ventricular assist devices, and—at long last—Food and Drug Administration–licensed benznidazole and nifurtimox, today, Chagas disease is still unknown to doctors and patients in countless places where infected people live.

Enter author Daisy Hernandez, an impassioned advocate for immigrants. Hernandez grew up watching her Colombian-born aunt slowly die of *T. cruzi*’s pitiless assault on both her heart and intestine.

“The doctors have sewn a line of dark stars across Tia Dora’s belly. Las cicatrices. And they have told her a word my mother whispers when she thinks I am not listening. Chagas.”

Thus reads an early passage in Hernandez’s exceptional book, *The Kissing Bug—A True Story of a Family, An Insect, and a Nation’s Neglect of a Deadly Disease*. Parenthetically, does anyone seem to care that Daisy has not yet started first grade when viewing Tia Dora’s colostomy bag or serving as her medical translator during a month-long stay at Columbia Presbyterian Hospital? Evidently not, because what other choice does the family have?

For much of her childhood, Daisy’s horror of Chagas was largely focused on *bichas*—one of several Spanish words for *T. cruzi*’s triatomine vectors.

“The women in my family constructed a private mythology. While other girls of my age were taught to fear rabid dogs and horrible men, I learned to be terrified of an insect the size of my fingernail, an insect that could kill a woman’s heart” her grown-up self writes. But a few years after Tia Dora’s death, as a newly-minted journalist, Daisy is finally interrogating the history, biology, and appearance of kissing bugs (“a cross between a cucaracha and a beetle”)—visiting an *insectario* at the University of the Andes in Bogota—even unearthing a chilling experiment published in 1943 in the *American Journal of Tropical Medicine*. In it, a researcher inoculated the eye of an inmate at the Texas State Lunatic Asylum with crushed kissing bug body parts, and, weeks later, observed parasites “vibrating” in the African-American man’s blood.

Hernandez’s book not only looks back, but also covers cutting-edge social and epidemiological aspects of Chagas disease: its current diasporic spread; its Southwest beachheads revealed in autochthonous cases both in humans and (in Texas, in particular) military and shelter dogs; its new ecological niches possibly related to clearing land for housing; the United States’ woefully inadequate screening of high-risk people and use of drugs to prevent long-term complications and congenital infections; even modern cardiac care and heart transplants as ultimate salvage procedures. If only so many modern Miguels were not hidden in “a Second America,” that is.

It’s “an American reality,” Hernandez adds for good measure in a closing chapter of *The Kissing Bug*, that “some people are taken care of and others are not.”

For more first-hand experience caring for immigrants with Chagas disease, I spoke with cardiologist Sheba Meymandi, a University of California Los Angeles (UCLA) professor of medicine and director of the Center of Excellence for Chagas disease at L.A. County’s Olive View–UCLA Medical Center. Since opening in 2007, the Center has diagnosed and treated over 350 patients. For years, Meymandi and her staff also met with local high-risk communities to conduct free medical exams, educate, and screen blood to detect *T. cruzi* infections and initiate treatment.

## INTERVIEW WITH DR. SHEBA MEYMANDI

### Let’s begin with your connection to Daisy Hernandez’s family and how you launched the Center of Excellence for Chagas Disease at Olive View–UCLA Medical Center.

This first answer is easy. After Tia Dora passed, Daisy’s sister Liliana sent me a lovely letter and a check for $1,500. She donated the money because we were the only clinical center in the country focused on Chagas. I was so touched I kept the letter. Periodically, I still look at it because it reminds me why we do what we do.

As for starting the clinic, like all of us, in medical school, I might have had an hour on Chagas, and I always thought of it as an exotic fascinoma. But in 2001, Dr. Glenn Mathisen, my infectious diseases colleague at Olive View said, “Let’s do a little study in the heart failure population and see what our prevalence [of Chagas disease] is.” In 2001, it was slightly less than 5%. In 2007, when treatment with benznidazole became available through CDC, the prevalence was 19%. In our patients with bundle branch blocks and our pacer population, the prevalence was 5% and 7%.

Around the same time, I started our outreach project using the county van and got EKGs and echocardiograms in addition to *T. cruzi* titers, which was labor intensive but also fun, because everyone wanted the EKG and the echo. We used to screen 100 or 110 people at each outreach. But after looking at a year’s worth of data, we saw that the EKG and the echo didn’t really add much, so we scaled back to just getting the titers in addition to cholesterol and diabetes screening.

### So then you did confirmatory tests and started treating patients, right?

Yes. When we first started, Vaughn Kirchhoff at the University of Iowa did all of our serology. Then the American Red Cross started to refer their positive blood donors to our Center for confirmatory testing, and the CDC took over the laboratory testing.

As for treatment, in the beginning I had no experience whatsoever and I called Sue Montgomery at CDC, who was very supportive. I also consulted people in South America about treatment [which was given over two to three months and often triggered side effects]. Honestly, I didn’t get a ton of guidance because I don’t think people really knew. It was kind of like shooting from the hip. If someone’s not tolerating the drug, decrease the dose, extend the duration, et cetera, et cetera.

### Please tell us about your local team.

First there was Jenny Rosales, who I call “Mama Jenny,” one of my cardiac cath nurses who later retired. Without her, we would never have gotten off the ground because she had the connection with Holy Cross Parish nurses, the *promotoras*. Mama Jenny was also the one who got us into health fairs and brought PVC piping so we could hang drapes and create rooms for our echos and EKGs.

By the way, the churches were hugely important in terms of patients trusting us, because if the churches trusted us, that meant we were legitimate and we wanted to help. Churches would even announce at masses: “They’re here to do screenings for Chagas, which can affect your heart. Please go get screened.” So, for the most part, when we called and said, “Look, you have Chagas; we’d like to bring you in for treatment,” patients would come in and have a conversation with us.

Then Jenny brought me Salvador Hernandez [no relation to Daisy], who was a green foreign medical grad from Guadalajara, and said “Shevita, you need to hire him.” So we hired him and started our clinical research trials. Today, he’s completing his cardiology fellowship at Kaiser and he’s a co-author on roughly 20 papers from our Center.

### Please talk about your personal connection to immigrants as well as your own medical history.

Although I was born and raised in the U.S., I’m first-generation Iranian-American. So this is another connection with Daisy Hernandez’s book. My mother has a heavy accent, and we were very poor, and I saw how my mom was treated. Being poor in this country is not easy. That’s why I ended up working for the L.A. County Health Department. Not for a second did I ever want a job in private practice. I’ve been very fortunate and blessed to work for the County and, for me, the Chagas work represents the epitome of preventive medicine. Let’s catch it early and treat it.

My own history? I was age 17 when I developed Type 1 diabetes following a viral infection. Fortunately, by then, I had good health insurance. But I was very ill, very brittle. At one point I couldn’t hold down food. I was down to 89 pounds and I was hospitalized for 3 months. Today I have various medical issues. So I have a lot of empathy for others who are very sick and stuck in the hospital or don’t have advocates.

### I’ve always been impressed with the exceptional quality of care you’ve managed to deliver to your county hospital patients with Chagas.

When I started our clinic, the hospital administrator was very supportive. I was also aggressive with our population because I had the gut feeling that—if we caught people early— we’d have an impact. One of our earliest patients was so scared of her diagnosis and being labeled with Chagas that she refused treatment. Within the year, she developed significant cardiomyopathy and ultimately arrested and died because she was fearful. I’ve never seen such an accelerated course. That was very scary for me because we tend to think of Chagas as a chronic disease which produces gradual dilatation of the left ventricle. But in Elena’s case, her ejection fraction went from 60 to 20% in a year.

Cardiomyopathy is one of the more expensive Medicare diagnosis-related groups [DRGs] and it’s also probably one of the more lethal DRGs. We also knew that some of our patients had really ugly ventricular arrhythmias. So here’s what we do now. If we see that a patient’s left ventricular size is dilated or the function is even a little bit off, first of all, we always get a cardiac MRI looking for delayed enhancement. If we find evidence of that, even if their ejection fraction is practically normal, we proceed to an electrophysiologic study [EPS] to see if we can elicit ventricular arrhythmias. Any patient with a positive EPS and any patient with cardiomyopathy gets a defibrillator.

And this is anecdotal, but at least 3 patients out of less than 200 have had a significant arrest within three months of getting their device. This encouraged us to add the device to our protocol. There are a lot of richer practices in South America—and also universities—that do this. But this is what we provide to our county patients at Olive View who have no funding. Now that the U.S. has more of a true Chagas network, it gives me a sense of pride that I’ve been able to share our protocols and have a broader impact.

### Final question. In addition to better screening of high-risk populations for Chagas disease, what’s on your wish list for the future and what will you do after you retire?

Well, just to be clear, my main wish—in fact, my number-one through number-ten wish—is that we do appropriate screening for Chagas disease. Here in the U.S., we aren’t even close.

A more academic interest—and I’ve tried to approach some UCLA colleagues about this—involves testing relatively young people who have sudden death. We all either know someone or have heard of someone who is an athlete and experiences a sudden-death cardiac arrest. And they do the workup and there’s no cardiac abnormalities, no genetic abnormalities, no hypertrophic cardiomyopathy, no LDL [low-density lipoprotein] syndrome. I think it would be really fascinating to have a registry and get Chagas titers on these people.

After I stop clinical work with the County, I still want to do global work involving Chagas. For example, Portugal has a 42% increase in residents who are natives of Brazil; some of them undoubtedly carry *T. cruzi*. In any event, I will continue providing service to lower- and middle-income countries that don’t have top-notch medical care. Whether that care involves Chagas or non-communicable diseases like hypertension or diabetes, I don’t yet know, but humanitarian work is where I find true gratification.

## FURTHER READING
